# Screen use, sleep duration, daytime somnolence, and academic failure in school-aged adolescents

**DOI:** 10.1371/journal.pone.0281379

**Published:** 2023-02-14

**Authors:** Daniel Pérez-Chada, Sergio Arias Bioch, Daniel Schönfeld, David Gozal, Santiago Perez-Lloret, On behalf of the Sleep in Adolescents Collaborative study group

**Affiliations:** 1 Pulmonary Medicine, Universidad Austral, Hospital Universitario Austral, Pilar, Argentina; 2 Instituto Nacional de Enfermedades Respiratorias “Dr. Emilio Coni”, Administración Nacional de Laboratorios e Institutos de Salud “Dr. Carlos G. Malbrán”, Santa Fe, Argentina; 3 Centro Diagnóstico San Jorge, Puerto Madryn, Chubut, Argentina; 4 Department of Child Health and Child Health Research Institute, University of Missouri School of Medicine, Columbia, Missouri, United States of America; 5 Consejo Nacional de Investigaciones Científicas y Técnicas (CONICET), Buenos Aires, Argentina; 6 Observatorio de Salud Pública, Pontificia Universidad Católica Argentina, Buenos Aires, Argentina; 7 Faculty of Medicine, Department of Physiology, University of Buenos Aires, Buenos Aires, Argentina; Dalhousie University, CANADA

## Abstract

In this study, we examined the relationship between screen time use, sleep characteristics, daytime somnolence, and academic performance in school-aged adolescents. We surveyed 1,257 12- to 18-year-old adolescents attending 52 schools in urban or suburban areas of Argentina. We recorded the daily exposure to various screen-based activities, including video- and online-gaming, social media, TV or streaming. Screen time and device type in the hour before bedtime, sleep patterns during weekdays and weekends, somnolence (Pediatric Daytime Sleepiness Scale score), and grades in language and mathematics were also assessed. Structural Equation Modelling was used to identify a path connecting the latent variables. Results are expressed as standardized regression weights (srw). Missing data were present in 393 subjects, and thus the final sample consisted of 864 complete responses. Daytime somnolence (i.e., PDSS score ≥ 15) was observed in 614 participants (71%), and academic failure (i.e., grades < 7/10) in 352 of them (41%). Time spent using video gaming consoles was negatively associated with sleep duration (srw = -0.22, p<0.01) and positively connected with daytime somnolence (srw = 0.11, p<0.01). Use of mobile devices was associated with lower academic performance (srw = -0.11, p<0.01). Sleep duration was inversely related to daytime somnolence (srw = -0.27, p<0.01), which was in turn negatively associated with academic performance (srw = -0.18, p<0.05). Bedtime computer use did not influence any outcome. In summary, among adolescents, screen use adversely affected nighttime sleep, daytime somnolence, and academic performance. These findings call for the implementation of educational public campaigns aimed at promoting healthy sleep and reducing screen exposure among adolescents.

## Introduction

Technological advances over the last decades have markedly enhanced the use of screen devices for both work and leisure activities (e.g., gaming, social networking, or watching multimedia contents) at all ages. For example, the proportion of 0 to 8-year-old children that had access to mobile devices grew from 52% in 2011 to 75% in 2013 [1]. Some studies suggest that school-aged children and adolescents spend about 7 hours per day in front of a screen (i.e., about 43% of the waking day assuming 8 hours of sleep per night) [2,3]. In many instances, screens are used as “babysitters”, allowing parents to conduct work activities at home or perform their domestic chores [4].

Excessive screen use may affect overall health, disrupt sleep, and reduce learning and academic performance. One of the most obvious effects of screen use is the reduction in physical activity, thereby leading to sedentarism and facilitating weight gain [5–7]. This is further aggravated by changes in diet related to advertising campaigns, and also spurred by behaviors associated with screen use, such as snacking [8–11]. Obesity and sedentarism, in turn, increase the risk of hypertension and other metabolic disorders, while further perpetuating the unhealthy behaviors that promoted their occurrence [6].

Sleep time and quality are also affected by screen use [2,12]. Time spent in front of the screen usually comes at the expense of sleep duration [13]. In addition, bright light and electromagnetic radiation, when used during the evening, inhibit melatonin production [13,14], thereby altering circadian rhythms. Furthermore, psychophysiological arousal incited by video gaming or social media use, particularly before bedtime, may impair pre-sleep relaxation, and further delay sleep onset [13,15].

Sleep is essential for learning [16], as it participates in the consolidation of declarative and procedural memories [17,18]. The reactivation during sleep of memories acquired during wakefulness is fundamental for consolidation of such memories in brain regions such as the hippocampus [19,20]. Regular sleep facilitates key aspects of synaptic plasticity, whereas extended wakefulness tends to reduce it [21]. It is thus not surprising that academic performance may be impaired among children and adolescents with poor sleep quality, as shown in a recent meta-analysis involving 13,631 school-aged children and adolescents participating in 16 different studies [22]. Daytime sleepiness resulting from insufficient sleep also imposes a profound adverse impact on learning and academic performance [22–24].

A recent meta-analysis involving 480,479 participants aged 4 to 18 years, showed that television viewing and video gaming were associated with reduced academic performance [25]. It is tempting to hypothesize that sleep disturbances and daytime somnolence mediate the deleterious effects of screen use on academic performance. A recent study in more than 10,000 children showed that adequate sleep time and screen use ≤ 2-h daily independently predicted improved academic performance [26]. Notwithstanding, the interaction(s) between these factors remains to be explored.

In this study, we explored the relationship between screen time use, sleep characteristics, daytime somnolence, and academic performance in a sample of school-aged adolescents from Argentina.

## Methods

### Sample population

Twelve- to eighteen-year-old adolescents attending the morning shift of participating schools were invited to answer a survey between May and November 2019. Fifty-two schools, both urban and suburban, participated in the survey.

### Procedures

The protocol was approved by the Institutional Review Board of the Austral University (Buenos Aires, Argentina, CIE17-044-B). Participants were requested to complete the questionnaire in their smartphones or in computers provided by the schools after written consent was obtained from the parents or caretakers, as well as from the school authorities. Students were free to participate or not at their own discretion.

After the survey was completed, we approached a subset of the participants and their corresponding schools (i.e., those that indicated the ability to provide grades), and asked for authorization to collect grades for language and mathematics achieved by each student in the trimester corresponding to the survey responses.

### Measurements

Daytime somnolence was assessed using the Pediatric Daytime Sleepiness scale [23,27]. Briefly, the PDSS is an 8-item questionnaire assessing the weekly frequency of episodes of inattention in classroom due to sleepiness, of sleep episodes while doing homework, sustaining adequate level of attention in the classroom, feeling tired or being in a bad mood during the day, problems for getting up from bed during the morning, falling asleep immediately after being awakened, needing someone to wake you up in the morning, or feeling the need of more sleep. It has a Chronbach alpha = 0.74 [23]. PDSS scores > 15 indicate the presence of daytime somnolence [23,27]. Sleep patterns were obtained by collecting information on bedtimes and wake up times during weekdays and weekends, whether any naps were obtained during weekdays and weekends and their duration, and dinner times during weekdays. In addition, distance from home to school, and information about the composition and characteristics of the household were collected.

Weight and height were inquired, and Body Mass Index was calculated and then transformed to age- and gender-adjusted Z-scores. Overweight and obesity were defined as Z-scores > 1 or > 2, respectively [28].

Exposure times spent with various types of screens as related to specific activities were recorded, including video gaming devices, tablets/smartphones for online gaming, social media networking, and watching TV or video streaming. Participants were instructed to document the average time spent per day engaged in each of these activities and deemed representative of the whole week. Answers were capped at 12-h, with higher values treated as missing data. Participants were also requested to indicate how many days per week computers, smartphones, tablets, gaming devices, or TV/streaming apps were used in the hour before bedtime.

Academic failure was defined as language and/or math grades < 7/10, as this is the cut-off point usually used in Argentina’s educational system for passing an exam.

### Statistical analysis

Numerical and categorical variables were compared between participants with or without daytime somnolence or according to academic results (i.e., failure or success) using t-tests (with an adjustment in cases where variances were non-homogeneous among the groups) or Chi-square tests, respectively. Data are presented as mean ±SD or frequencies as appropriate. Multivariate models were built using logistic regression. Only variables attaining statistical significance in bivariate comparisons, or those deemed of clinical relevance, were included in the multivariate models. Multicolinearity was absent from all models. A likelihood ratio test was used to test whether the relationship between screen time variables and outcomes (i.e., daytime somnolence and academic failure) was of a linear or non-linear nature. All statistical tests were conducted using statistical software SPSS v.23 (IBM®, Chicago, Ill). A two-tailed p value designating statistical significance was set at <0.05.

### Structural equation modelling analysis

A complex multi-step pathway connecting screen use, sleep time, daytime somnolence, and academic performance was modelled using Structural Equation Modelling (SEM) [24]. We followed the usual procedures [29]. Briefly, the model was initially fitted by using the Maximum Likelihood estimation (ML) method. Univariate and multivariate normality was assessed by computing Z-tests on variable’s kurtosis. Variable’s kurtosis > 5 or multivariate kurtosis critical value > 7 were considered as indicators of non-normality [29]. Variables with kurtosis > 5 were log10-transformed and multivariate kurtosis was rechecked. If non-normality was still detected, the model was refitted by using the ML method with 100-sample bootstrapping. The factor structure of the PDSS and of the latent variable “academic failure” were assessed in first place by Confirmatory Factor Analysis (CFA). Latent variables connected with screen use and sleep characteristics were explored, first by exploratory factor analysis, and then tested by CFA. Model’s validity was assessed by the Chi-square (χ2) statistic, the root mean square error of approximation (RMSEA), the Comparative Fit Index (CFI), and Normed Fit Index (NFI). Only after a satisfactory measurement model was built (i.e., RMSEA < 0.08 and CFI/NFI>0.70), we fitted the path model connecting the latent variables (i.e. sleep duration, daytime somnolence, 0-to-10 math and language grades to assess academic performance, and screen use) between each other.

We used Modification Indices (MI) to modify the model by including significant associations not modelled before, if they had a biological or clinical significance. Results from SEM are presented in terms of standardized regression weights (srw) between pairs of variables. Such coefficients represent the strength of the relationship between any pair of variables independently from confounding factors. P-values < 0.05 were considered statistically significant. SEM analysis was performed with AMOS v.23 (IBM®, Chicago, Ill).

## Results

A total of 1,257 subjects participated in the survey and provided academic grades. Of these, 393 participant responses had missing data (299 had missing data on sleep variables and 132 had missing data on screen use). Such that the final sample was comprised of 864 subjects. The characteristics of the participants are shown in Table 1. Compared to those that were included in the final sample, participants with missing data had lower average grades (7.40±1.37 vs 7.09±1.56, respectively, p<0.01), and had a higher frequency of academic failure (40.7% vs 48.1%, respectively, p = 0.01).

**Table 1:** Characteristics of participants with or without daytime somnolence.

	Whole Sample (n = 864)	No daytime somnolence (n = 250)	Daytime somnolence (n = 614)	p-value	Model 1 OR (95% CI)	Model 2 OR (95% CI)
Age (y)	15.5±1.6	15.3±1.7	15.5±1.5	0.08		
Male gender	541 (63%)	139 (56%)	402 (65%)	<0.01	0.70 (0.52–0.95)*	0.68 (0.47–0.99)*
Parent’s educative school						
Elementary	11 (1%)	5 (2%)	6 (1%)	0.10		
High school	132 (16%)	46 (19%)	88 (14%)			
College/University	708 (83%)	194 (79%)	514 (85%)			
Nb. of rooms per household	5.7±2.1	5.6±2.0	5.8±2.1	0.22		
Nb. of sleep rooms per household	3.1±1.0	3.1±0.9	3.2±1.0	0.11		
Persons per household	3.6±1.6	3.5±1.5	3.6±1.6	0.30		
BMI (kg/m^2^)	20.7±2.9	20.6±3.0	20.7±2.9	0.87		
Underweight	13 (2%)	4 (2%)	9 (2%)	0.88		
Normal	485 (80%)	130 (78%)	355 (81%)			
Overweight	83 (14%)	24 (14%)	59 (13%)			
Obese	24 (4%)	8 (5%)	16 (4%)			
Parental education						
Elementary	11 (1%)	5 (2%)	6 (1%)	0.10		
High school	132 (16%)	46 (19%)	86 (14%)			
College/University	708 (83%)	194 (79%)	514 (85%)			
Sleep patterns during weekdays						
Dinner time on weekdays (h)	21.6±0.8	21.5±0.8	21.6±0.8	0.10	-	-
Time of going to bed (h)	23.4±1.1	23.2±1.0	23.5±1.2	<0.01	-	-
Sleep onset latency (min)	20.5±20.9	18.9±20.5	21.2±21.1	0.14	1.00 (0.99–1.01)	1.00 (0.99–1.01)
Sleep time (h)	7.0±1.2	7.2±1.2	7.0±1.3	0.02	0.86 (0.75–0.97)*	0.91 (0.79–1.05)
Awakening time (h)	6.8±0.7	6.7±0.7	6.8±0.7	<0.01	-	-
Time spent travelling to the school > 60 min	88 (10%)	29 (11%)	59 (10%)	0.38	-	-
Nap time (h)	1.1±1.8	0.9±1.6	1.2±1.9	0.01	-	-
Sleep patterns during weekends						
Time of going to bed (hs)	1.4±2.0	1.2±1.9	1.5±2.0	0.02	-	-
Sleep time (h)	9.4±1.7	9.2±1.6	9.4±1.7	0.07	1.10 (1.01–1.21)*	1.15 (1.04–1.26)**
Awakening time (h)	10.8±1.8	10.3±1.7	10.9±1.8	<0.01	-	-
Nap time (h)	0.8±1.6	0.7±1.7	0.8±1.6	0.25		
Screen time (h)						
Video gaming devices	0.7±1.5	0.6±1.3	0.7±1.5	0.48	-	1.16 (1.01–1.34)*
Mobile devices for gaming	1.5±2.0	1.4±1.8	1.6±2.1	0.21	-	1.05 (0.96–1.14)
Social Networking	3.1±2.5	2.6±2.3	3.3±2.6	<0.01	-	1.06 (0.99–1.14)
TV or streaming	2.2±1.8	2.0±1.5	2.3±1.9	0.02	-	1.04 (0.94–1.14)
Use before bedtime (days/week)						
Computers	0.7±1.5	0.6±1.5	0.7±1.4	0.97	-	0.95 (0.86–1.06)
Smartphones	5.9±2.0	5.4±2.4	6.1±1.8	<0.01	-	1.15 (1.07–1.24)**
Tablets	0.3±1.1	0.3±1.2	0.3±1.1	0.63	-	0.97 (0.85–1.11)
Gaming devices	0.6±1.4	0.6±1.5	0.6±1.4	0.64	-	0.95 (0.84–1.09)
TV/streaming apps	2.6±2.6	2.5±2.6	2.7±2.6	0.31	-	1.00 (0.94–1.07)

Means ± standard deviations are shown for numerical variables.

* p<0.05

** p<0.01 (Wald test).

### Screen time, sleep characteristics, and daytime sleepiness

Mean PDSS score was 18.4±5.1, daytime somnolence (i.e., PDSS > 15) was present among in 614 (71%) of the responders. Statistically significant differences between somnolent and non-somnolent groups emerged for the frequency of male gender, weekday bedtime, sleep time, awakening time, and nap time, as well as weekend sleep patterns (Table 1). Among subjects with daytime somnolence, time spent on social networks was higher than in those without somnolence (3.3±2.6 *vs*. 2.6±2.3 h/day, p<0.01), whereas bedtime smartphone use was higher (6.1±1.8 vs 5.4±2.4 days/week). Likelihood ratio tests results supported a linear relationship between screen times and daytime somnolence scores (results not shown). A logistic regression analysis identified female gender, longer weekend sleep time, higher time spent video gaming, and bedtime smartphone use as factors significantly and independently associated with daytime somnolence (Table 1).

### Screen time and other factors related to academic performance

Overall language and math grades were 7.51±1.39 (min-max: 3–10) and 7.18±1.74 (2–10). Out of the 864 participants, 352 (40.7%) failed in language and/or math. Participants who failed in their exams were more frequently males, suffered more frequently from daytime somnolence, slept less during weekdays, made longer naps, and went to sleep later on weekends (Table 2). Regarding screen time, participants who failed their exams spent longer times playing videogames (0.9±1.6 *vs*. 0.6±1.3 h/day in those who succeeded, p<0.01), spent longer time watching TV or streamed contents (2.4±1.8 *vs*. 2.1±1.8 h/day, p = 0.03), and used gaming devices at bedtime more frequently (0.7±1.6 *vs*. 0.5±1.3, p = 0.02). The likelihood ratio suggested that a non-linear relationship existed between academic failure and using mobile devices for gaming (data not shown). A logistic regression analysis identified that the following factors were significantly and independently connected with academic failure: male gender, higher PDSS score, shorter weekdays sleep time, and time spent using mobile devices for gaming (Table 2).

**Table 2:** Characteristics of participants with academic success or failure.

	Academic success (n = 512)	Academic failure (n = 352)	p-value	Model 1 OR (95% CI)	Model 2 OR (95% CI)
Age (y)	15.4±1.6	15.6±1.6	0.18		
Male gender	348 (68%)	193 (55%)	<0.01	1.86 (1.39–2.48)**	1.69 (1.20–2.40)**
BMI (kg/m^2^)	20.6±2.8	20.8±3.0	0.40		
Underweight	6 (2%)	7 (3%)	0.35		
Normal	299 (82%)	186 (77%)			
Overweight	46 (13%)	37 (15%)			
Obese	12 (3%)	12 (5%)			
Parental education					
Elementary	8 (2%)	3 (1%)	0.31		
High school	72 (14%)	60 (17%)			
College/University	425 (84%)	283 (82%)			
Nb. of rooms per household	5.8±2.1	5.6±2.1	0.06		
Nb. of sleep rooms per household	3.2±1.0	3.2±1.0	0.97		
Persons per household	3.6±1.5	3.5±1.6	0.51		
PDSS score	18.1±5.2	18.9±4.8	0.02	1.04 (1.01–1.06)**	1.04 (1.01–1.07)*
Daytime somnolence (PDSS score ≥ 15)	351 (60%)	263 (63%)	0.05	-	-
Sleep patterns during weekdays					
Dinner time on weekdays (h)	21.5±0.8	21.7±0.9	<0.01	-	-
Time of going to bed (h)	23.4±1.1	23.5±1.2	0.02	-	-
Sleep onset latency (min)	20.9±22.2	20.0±18.9	0.54	0.99 (0.98–1.01)	1.00 (0.99–1.01)
Sleep time (h)	7.1±1.2	6.9±1.3	0.02	0.87 (0.77–0.98)*	0.86 (0.76–0.97)*
Awakening time (h)	6.8±0.6	6.8±0.8	0.38		
Time spent travelling to the school > 60 min	64.4±241.8	95.4±356.8	0.16		
Nap time (h)	0.9±1.5	1.4±2.2	<0.01	-	-
Sleep patterns during weekends					
Time of going to bed (h)	1.2±1.9	1.6±2.1	<0.01	-	-
Sleep time (h)	9.4±1.6	9.2±1.8	0.06	0.95 (0.87–1.03)	0.94 (0.87–1.03)
Awakening time (h)	10.7±1.7	10.9±1.9	0.17		
Nap time (h)	0.7±1.4	0.9±1.9	0.09		
Screen time (h)					
Video gaming devices	0.6±1.3	0.9±1.6	<0.01	-	1.05 (0.93–1.18)
Mobile devices for gaming	1.4±2.0	1.7±2.0	0.10	-	1 h/d: 1.47 (1.05–2.05)* 2 h/d: 1.77 (0.69–4.55) 3 h/d: 1.85 (1.05–3.27)* 4 h/d: 1.26 (0.74–2.17) ≥ 5 h/d: 1.03 (0.54–1.95)
Social Networking	3.0±2.5	3.2±2.5	0.40	-	1.00 (0.94–1.07)
TV or streaming	2.1±1.8	2.4±1.8	0.03	-	1.07 (0.98–1.16)
Use before bedtime (days/week)					
Computers	0.7±1.4	0.6±1.5	0.57	-	0.94 (0.85–1.04)
Smartphones	5.9±2.0	5.9±2.1	0.73	-	0.96 (0.89–1.03)
Tablets	0.3±1.2	0.3±0.9	0.42	-	0.92 (0.81–1.06)
Gaming devices	0.5±1.3	0.7±1.6	0.02	-	1.04 (0.92–1.17)
TV/streaming apps	2.6±2.5	2.7±2.6	0.46	-	1.03 (0.97–1.09)

Means ± standard deviations are shown for numerical variables. * p<0.05 ** p<0.01 (Wald test). Model 2 included all variables in Model 1 plus Screen times.

### A multi-step pathway connecting screen time, sleep patterns, daytime somnolence, and academic performance

The characteristics of the final measurement model are shown in Table 3. To achieve univariate and multivariate normality, several variables were log10-transformed, including weekly computer bedtime use, bedtime tablet use, and time spent video gaming. Sleep latency was initially included, but later left out, as it increased the multivariate non-normality ratio, and did not contribute to the predictive power of the model. In addition, 9 outlier cases were excluded based on the calculation of Mahalanobis squared distances. In the final model, all variables were normally distributed, and multivariate non-normality critical ratio was 7.9, slightly above the required level. Therefore, bootstrapping results are also provided in Table 3. MIs did not suggest any significant modification. The model included the following latent variables, which were included in the path diagram: Bedtime computer use (days/week), time spent using of video gaming consoles (h/day), Time spent using mobile devices (h/day), sleep duration, male gender, PDSS Somnolence score, and academic performance. The fit was fair, with CFI = 0.753, NFI = 0.728, RMSEA = 0.089, and AIC = 1637.27.

**Table 3:** Characteristics of the measurement model.

		ML estimation		Bootstrapping	
Latent Variable	Indicators	Regression weights	P-value	95% CI	P-value
Bedtime computer use	Bedtime computer use	1	-	-	-
Time spent using of video gaming consoles	Time spent video gaming	1	-	-	-
	Bedtime videogame console use	-0.303	<0.001	-0.303, -0.202	0.03
Time spent using mobile devices	Time spent watching TV online	0.198	<0.001	0.198, 0.332	0.004
	Time spent on social networks	0.278	<0.001	0.278, 0.434	0.138
	Time spent video gaming on mobile devices	1	-	-	-
	Bedtime Tablet use	1.966	<0.001	1.966, 5.120	0.017
Sleep duration	Weekdays sleep time	1	-		. . .
	Weekend’s sleep time	0.449	<0.001	0.449, 0.587	0.004
	Weekdays time of going to bed	-1.011	<0.001	-1.011, -0.963	0.013
	Weekend’s time of going to bed	-1.057	<0.001	-1.057, -0.982	0.005
Male gender	Gender	1	-	-	-
PDSS Somnolence score	sleep episodes while doing homework	1.006	<0.001	1.006, 1.126	0.043
	inattention in classroom due to sleepiness	-0.531	<0.001	-0.531, -0.450	0.034
	problems for getting up from bed during the morning	1.137	<0.001	1.137, 1.265	0.077
	falling asleep immediately after being awakened	1.247	<0.001	1.247, 1.386	0.133
	needing someone to wake you up in the morning	0.559	<0.001	0.559, 0.782	0.02
	feeling the need of more sleep	1.191	<0.001	1.191, 1.284	0.077
	sustaining adequate level of attention in the classroom	1	-	-	-
	feeling tired or being in a bad mood during the day	0.737	<0.001	0.608, 0.851	0.007
Academic performance	Language	1	-	-	-
	Mathematics	1.200	<0.001	1.200, 1.761	0.023

ML = Maximum Likelihood; CI = Confidence Interval. Regression Weights = 1 represent the anchors of the latent variables, which define their scales of measurement.

The characteristics of the final path model are shown in Table 4. The fit was still fair, with a CFI = 0.702, NFI = 0.679, RMSEA = 0.096, AIC = 1896.65. It should be kept in mind that the measurement model is more saturated as it allows for the correlation between all latent variables within each other, and thus may result in higher, yet spurious fit.

**Table 4:** Path diagram.

		ML estimation		Bootstrapping	
Dependent variable	Predictor	Regression weights	P-value	95% CI	P-value
Time spent using mobile devices	Male gender	-0.103	<0.001	-0.162, -0.039	0.03
	Time spent using of video gaming consoles	0.483	0.005	-1.491, -0.286	0.006
Sleep duration	Time spent using of video gaming consoles	-0.979	<0.001	0.687, 2.118	0.009
	Time spent using mobile devices	0.031	0.373	-0.057, 0.073	0.650
	Bedtime computer use	-0.012	0.620	-0.062, 0.029	0.647
Daytime somnolence	Male gender	0.05	<0.001	0.028, 0.079	0.009
	Time spent using of video gaming consoles	0.29	0.004	-0.649, -0.125	0.012
	Time spent using mobile devices	0.03	0.118	0.007, 0.065	0.033
	Bedtime computer use	0.013	0.429	-0.016, 0.039	0.480
	Sleep duration	-0.157	<0.001	-0.199, -0.120	0.005
Academic performance	Male gender	0.152	<0.001	0.059, 0.210	0.038
	Time spent using of video gaming consoles	0.043	0.745	-0.316, 0.171	0.732
	Time spent using mobile devices	-0.094	0.005	-0.191, -0.044	0.01
	Bedtime computer use	-0.012	0.660	-0.056, 0.042	0.709
	Sleep duration	0.017	0.692	-0.104, 0.096	0.819
	Daytime somnolence	-0.288	<0.001	-0.422, -0.124	0.030

ML = Maximum Likelihood; CI = Confidence Interval.

**Fig 1:**
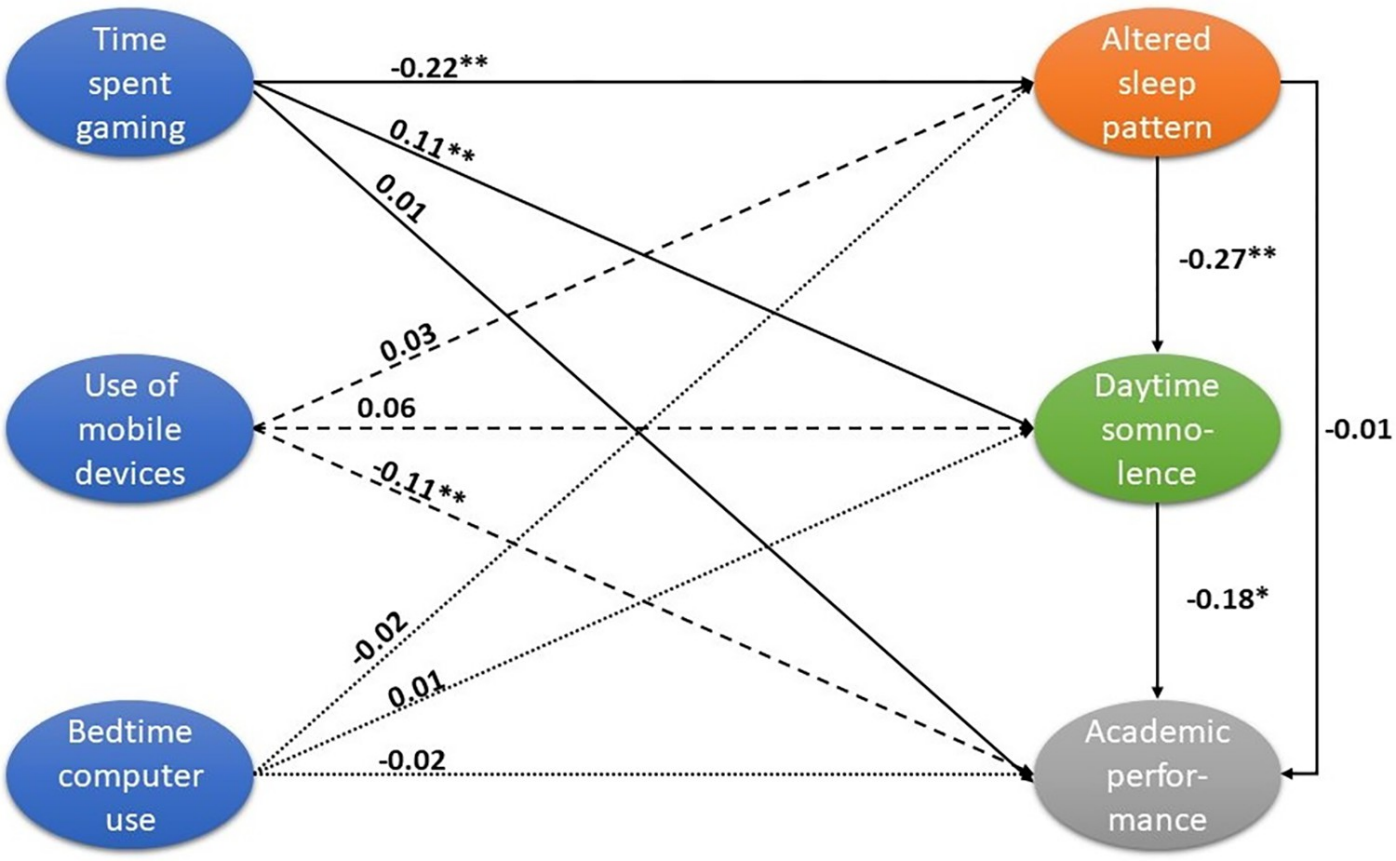
Relationship between screen use, sleep duration, daytime somnolence, and academic performance. Paths were fitted by Structured Equation modelling. Shown are Standardized Regression Weights which represent the strength of the relationship between any pair of variables independently from all other variables included in the model. * p<0.05, ** p<0.01.

The summary of the most relevant association between variables in the final path model is shown in Fig 1. Time spent using video gaming consoles was negatively related sleep duration (srw = -0.22, p<0.01) and positively connected with daytime somnolence (srw = 0.11, p<0.01). Use of mobile devices was associated with lower academic performance (srw = -0.11, p<0.01). Sleep duration was inversely related to daytime somnolence (srw = -0.27, p<0.01), which was in turn negatively associated with academic performance (srw = -0.18, p<0.05). Bedtime computer use did not influence any outcome. Sleep duration did not show a significant direct relationship with academic performance.

## Discussion

The present study including a large sample of 12- to 18-years old adolescents from large and small cities in Argentina, explores the direct and indirect burden imposed by screen use on daytime somnolence, and academic performance. The concurrent assessment of these two variables enabled improved modeling of the complex interrelationships between these outcomes. One of the findings is that the effects of screen time on these outcomes depended on the type of screen activity in which the participants were engaged and demonstrate that time spent video gaming affects sleep and increase daytime somnolence, independently from the effects of the former on the later. Using mobile devices was associated with worse academic performance, without effects on other outcomes. In agreement with our results, a recent meta-analysis involving 58 studies with a total of 480,479 participants showed that academic performance was significantly associated with watching TV or playing videogames, but not with the total daily time spent in front of screens.

Our results suggest that the effects of screen time on sleep duration and academic performance are related to the characteristics of each activity performed in front of screens. Use of mobile devices for watching online contents, social networking, or gaming, was associated with lower academic performance. The impact of screen use on learning is further stressed by the findings of altered brain structure in children and adolescents spending increased time in front of screens [30,31]. While it is tempting to conclude that withdrawing screens will directly result in academic performance improvements, further studies are needed to test the efficacy of this intervention.

Bedtime use of screens also adversely affected sleep, increased daytime somnolence, and reduced academic performance. Blue light emitted by screens suppresses melatonin production, causing circadian disruption and impairing sleep onset and continuity [13,14,32]. Impaired sleep is, in turn, the leading cause of daytime somnolence [23,24], which increases significantly the risk of academic failure [23,24]. Interestingly, it has been recently shown that an intervention directed at reducing the use of screens before bedtime was accompanied by earlier sleep onset times, increased total sleep duration, and improved daytime vigilance [33].

The strengths of our paper included a large sample size, in which screen use, sleep patterns, daytime somnolence, and academic performance were evaluated simultaneously, and a rigorous modelling of the interrelationship between these variables. Implementation of Structural Equation Modelling adds further insights since this analytical approach allows considering measurement errors when assessing the relationships between pairs of variables, which can be combined in a multi-step path. Our study has some limitations. Its cross-sectional nature and the absence of targeted interventions aimed at reducing specific screen times preclude the assessment of causality between screen time, somnolence, and academic performance. Our results are based on self-reported screen use. Participants may not recall such exposures with precision or may be unwilling to disclose them with accuracy. Indeed, some participants reported screen use beyond 12 hours per day, likely an exaggeration, and therefore were excluded from the data analysis. Errors in reporting might have increased data variability, thus obscuring some relationships, but bias is unlikely. Of note, a small but significant difference in academic performance was noted between participants with or without missing data, suggesting the possibility of a selection bias. Regarding the SEM model, caution is advised when interpreting the regression weights of the log10-transformed variables. However, the direction of the association and its statistical significance is still adequately represented.

## Conclusions

Adolescents who play video games for longer periods of time exhibit shorter sleep duration and higher probability of reporting daytime somnolence, whereas those that used mobile devices more extensively showed increased risk for reduced academic performance. In addition, reduced sleep duration was associated with daytime somnolence, which in turn adversely affected academic performance. Our findings highlight the importance of maintaining an adequate sleep hygiene and of limiting the use of screens to preserve learning among adolescents. Future studies should be designed while accounting for the findings reported herein.

## Supporting information

S1 File Contains all the supporting tables and figures. (DOC)
