# Supplementary material for: Screen use, sleep duration, daytime somnolence, and academic failure in school-aged adolescents
Source: PLoS One. 2023 Feb 14;18(2):e0281379. doi: 10.1371/journal.pone.0281379 (PMC9928097; doi:10.1371/journal.pone.0281379)
Supplement: S1 File — (DOC) [file pone.0281379.s001.doc]

Table E-1: Matrix of inter-item correlation for the variables included in the logistic regression análisis

|  | Constant | Gender | Sleep lat | Sleep time week | Sleep time weekend | PDSS | Videogaming time | S.Network time | TV time | Computer use | Cellphone time | Cellphone use | Tablet use | Videogames use | TV use |
| --- | --- | --- | --- | --- | --- | --- | --- | --- | --- | --- | --- | --- | --- | --- | --- |
| Constant | 1,000 | -,184 | -,142 | -,612 | -,479 | -,316 | ,003 | -,174 | -,072 | -,041 | -,016 | -,372 | ,057 | ,116 | ,017 |
| Gender | -,184 | 1,000 | -,003 | -,042 | ,103 | ,064 | -,344 | ,193 | ,051 | ,003 | -,055 | ,051 | ,036 | -,240 | ,069 |
| Sleep lat | -,142 | -,003 | 1,000 | ,167 | -,069 | -,032 | ,026 | -,002 | ,001 | -,049 | ,018 | -,040 | ,001 | -,035 | -,007 |
| Sleep time week | -,612 | -,042 | ,167 | 1,000 | -,200 | ,067 | ,002 | ,151 | ,037 | ,077 | -,077 | ,123 | -,088 | -,064 | -,070 |
| Sleep time weekend | -,479 | ,103 | -,069 | -,200 | 1,000 | -,100 | -,024 | ,058 | -,036 | ,006 | ,061 | ,120 | ,010 | -,045 | -,011 |
| PDSS | -,316 | ,064 | -,032 | ,067 | -,100 | 1,000 | -,010 | -,043 | -,031 | -,012 | -,035 | -,152 | -,033 | ,016 | ,000 |
| Videogaming time | ,003 | -,344 | ,026 | ,002 | -,024 | -,010 | 1,000 | -,047 | -,113 | -,138 | -,002 | ,039 | ,020 | -,355 | ,158 |
| S.Network time | -,174 | ,193 | -,002 | ,151 | ,058 | -,043 | -,047 | 1,000 | -,242 | ,063 | -,183 | -,167 | ,091 | -,046 | -,022 |
| TV time | -,072 | ,051 | ,001 | ,037 | -,036 | -,031 | -,113 | -,242 | 1,000 | -,006 | -,088 | ,018 | -,097 | ,044 | -,145 |
| Computer use | -,041 | ,003 | -,049 | ,077 | ,006 | -,012 | -,138 | ,063 | -,006 | 1,000 | -,029 | -,122 | -,007 | -,003 | -,056 |
| Cellphone time | -,016 | -,055 | ,018 | -,077 | ,061 | -,035 | -,002 | -,183 | -,088 | -,029 | 1,000 | ,028 | -,119 | -,035 | -,013 |
| Cellphone use | -,372 | ,051 | -,040 | ,123 | ,120 | -,152 | ,039 | -,167 | ,018 | -,122 | ,028 | 1,000 | -,011 | -,067 | -,172 |
| Tablet use | ,057 | ,036 | ,001 | -,088 | ,010 | -,033 | ,020 | ,091 | -,097 | -,007 | -,119 | -,011 | 1,000 | -,148 | -,047 |
| Videogames use | ,116 | -,240 | -,035 | -,064 | -,045 | ,016 | -,355 | -,046 | ,044 | -,003 | -,035 | -,067 | -,148 | 1,000 | -,226 |
| TV use | ,017 | ,069 | -,007 | -,070 | -,011 | ,000 | ,158 | -,022 | -,145 | -,056 | -,013 | -,172 | -,047 | -,226 | 1,000 |

Encuesta sobre hábitos de sueño en estudiantes jóvenes.

**CÓDIGO**

|  |  |  |  |  |  |  |  |  |  |  |  |  |  |  |  |  |
| --- | --- | --- | --- | --- | --- | --- | --- | --- | --- | --- | --- | --- | --- | --- | --- | --- |

**CARACTERISTICAS SOCIODEMOGRÁFICAS**

¿Cuál es el máximo nivel educativo alcanzado por tu madre, padre o tutor?:

| Sin educación |  |
| --- | --- |
| Primario incompleto |  |
| Primario completo |  |
| Secundario incompleto |  |
| Secundario completo |  |
| Terciario o universitario incompleto |  |
| Terciario o universitario completo |  |
| No sabe |  |

¿Cuántos ambientes/habitaciones tiene el hogar donde vivís? (sin contar cocina y baño) _____ambientes

¿Cuántos de estos ambientes/habitaciones se usan para dormir? _____ ambientes

¿Cuántas personas además de vos viven en el hogar? ______ personas

**CARACTERÍSTICAS DEL SUEÑO**

¿Con qué frecuencia sientes tanto sueño que te cuesta prestar atención en la clase?

Siempre (todos los días)

Frecuentemente (3 o más veces por semana)

Casi nunca (menos de 1 vez por semana)

A veces (1 o 2 veces por semana)

Nunca

¿Con qué frecuencia te quedas dormido/a o te da sueño mientras haces la tarea?

Siempre (todos los días)

Frecuentemente (3 o más veces por semana)

Casi nunca (menos de 1 vez por semana)

A veces (1 o 2 veces por semana)

Nunca

¿Estás atento/a o alerta en clase?

Siempre (todos los días)

Frecuentemente (3 o más veces por semana)

Casi nunca (menos de 1 vez por semana)

A veces (1 o 2 veces por semana)

Nunca

¿Con qué frecuencia te sientes cansado y de mal humor durante el día?

Siempre (todos los días)

Frecuentemente (3 o más veces por semana)

Casi nunca (menos de 1 vez por semana)

A veces (1 o 2 veces por semana)

Nunca

Siempre (todos los días)

Frecuentemente (3 o más veces por semana)

Casi nunca (menos de 1 vez por semana)

A veces (1 o 2 veces por semana)

Nunca

Siempre (todos los días)

Frecuentemente (3 o más veces por semana)

Casi nunca (menos de 1 vez por semana)

A veces (1 o 2 veces por semana)

Nunca

Siempre (todos los días)

Frecuentemente (3 o más veces por semana)

Casi nunca (menos de 1 vez por semana)

A veces (1 o 2 veces por semana)

Nunca

Siempre (todos los días)

Frecuentemente (3 o más veces por semana)

Casi nunca (menos de 1 vez por semana)

A veces (1 o 2 veces por semana)

Nunca

¿Te cuesta levantarte de la cama por la mañana?

¿Vuelves a quedarte dormido/a después de que te despertaron por la mañana?

¿Necesitas que alguien te despierte por la mañana?

¿Con qué frecuencia sientes necesidad de dormir más tiempo?

______ : ______

Horas Minutos

______ : ______

Horas Minutos

______ : ______

Horas Minutos

______ : ______

Horas Minutos

______ : ______

Horas Minutos

______ : ______

Horas Minutos

¿Cómo viajas al colegio la mayoría de las veces?

¿Cuánto tiempo demoras desde que sales de tu casa hasta que llegas al colegio? (indica en horas, minutos)

¿Cuánto tiempo duermes la siesta los días de semana? (si no duermes siesta marca 00:00)

¿Cuánto tiempo duermes la siesta los fines de semana o feriados? (si no duermes siesta marca 00:00)

¿Cuánto tiempo tardas en dormirte?

Auto

Caminando

Transporte escolar

Transporte público (colectivo, tren, otro)

En bicicleta

Otros

¿A qué hora te acuestas los días de semana?

¿A qué hora cenas durante los días de semana?

______ : ______

Horas Minutos

______ : ______

Horas Minutos

______ : ______

Horas Minutos

______ : ______

Horas Minutos

______ : ______

Horas Minutos

______ : ______

Horas Minutos

______ : ______

Horas Minutos

______ : ______

Horas Minutos

¿A qué hora te levantas los días de semana?

¿A qué hora te acuestas los fines de semana?

¿A qué hora te levantas los fines de semana?

**Habitualmente, fuera del horario escolar y durante la semana.**

**¿Cuánto tiempo le dedicas por día a los siguientes juegos o actividades? (si no lo haces indica tiempo “00:00”)**

Videojuegos en computadora o consolas (PlayStation, Xbox, WII, etc.).

Juegos en tablet y/o teléfono celular.

____ Horas: ____ Minutos

Uso de redes sociales (facebook, instagram, twitter, snapchat, etc.)

Mirar televisión o contenido online (TV, Netflix, youtube, etc.)

Otras actividades frente a una pantalla.

______ : ______

Horas Minutos

______ : ______

Horas Minutos

**En tu dormitorio y durante la última hora antes de dormirte.**

**¿Con qué frecuencia usas los siguientes dispositivos electrónicos?**

PC o laptop

Teléfono celular

______ : ______

Horas Minutos

______ : ______

Horas Minutos

______ : ______

Horas Minutos

Consola de juegos

TV o Smart TV

Tablet


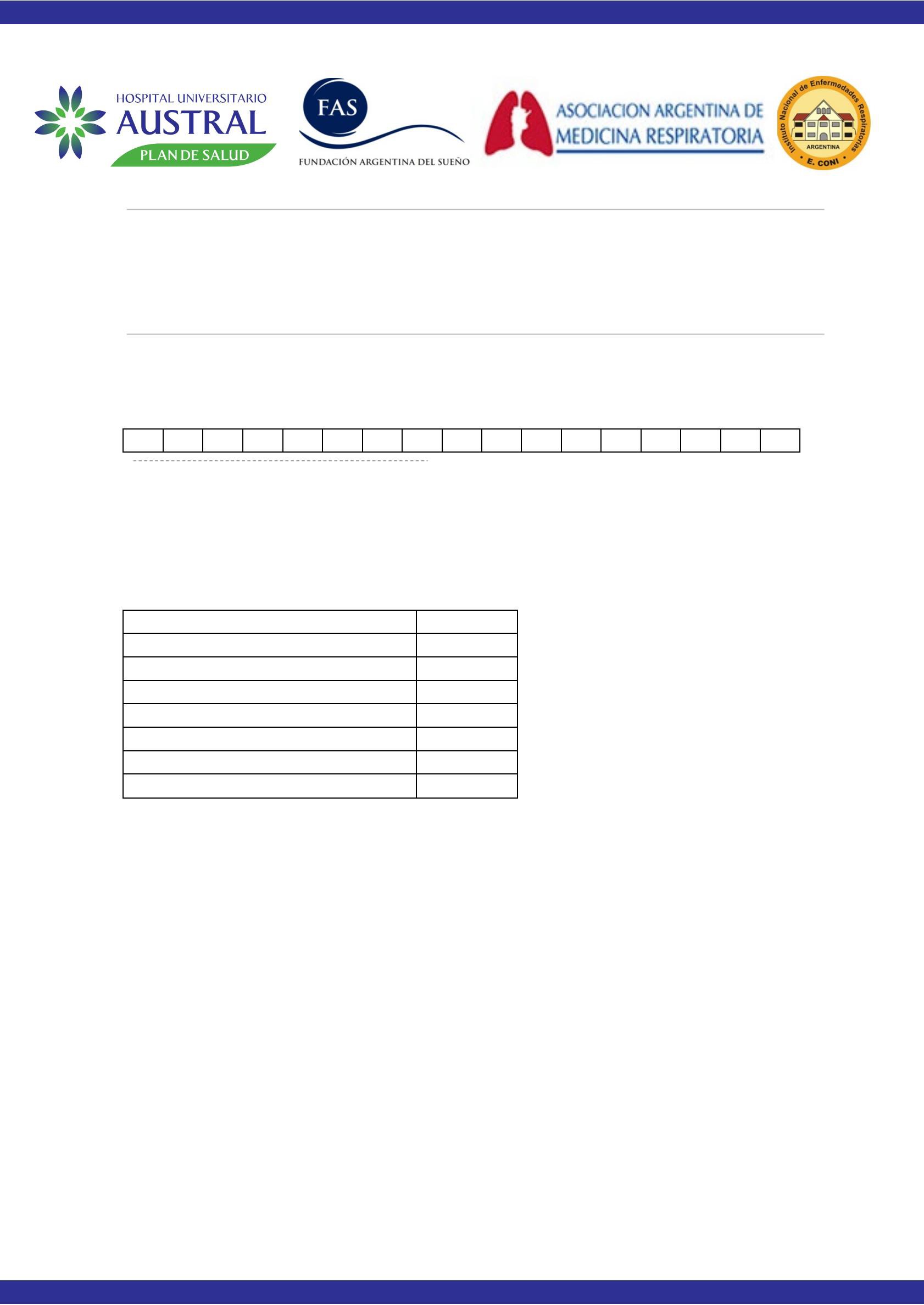


Survey on sleep habits in young students.

**CODE**

**SOCIODEMOGRAPHIC CHARACTERISTICS**

What is the highest educational level attained by your mother, father or guardian?:

Without education

incomplete primary complete primary incomplete secondary complete high school

Incomplete tertiary or university Completed college or university

Does not know

How many main rooms/rooms does the home where you live have? (not counting kitchen and bathroom) rooms

How many of these environments/rooms are used for sleeping? environments

How many people besides you live in the home? people


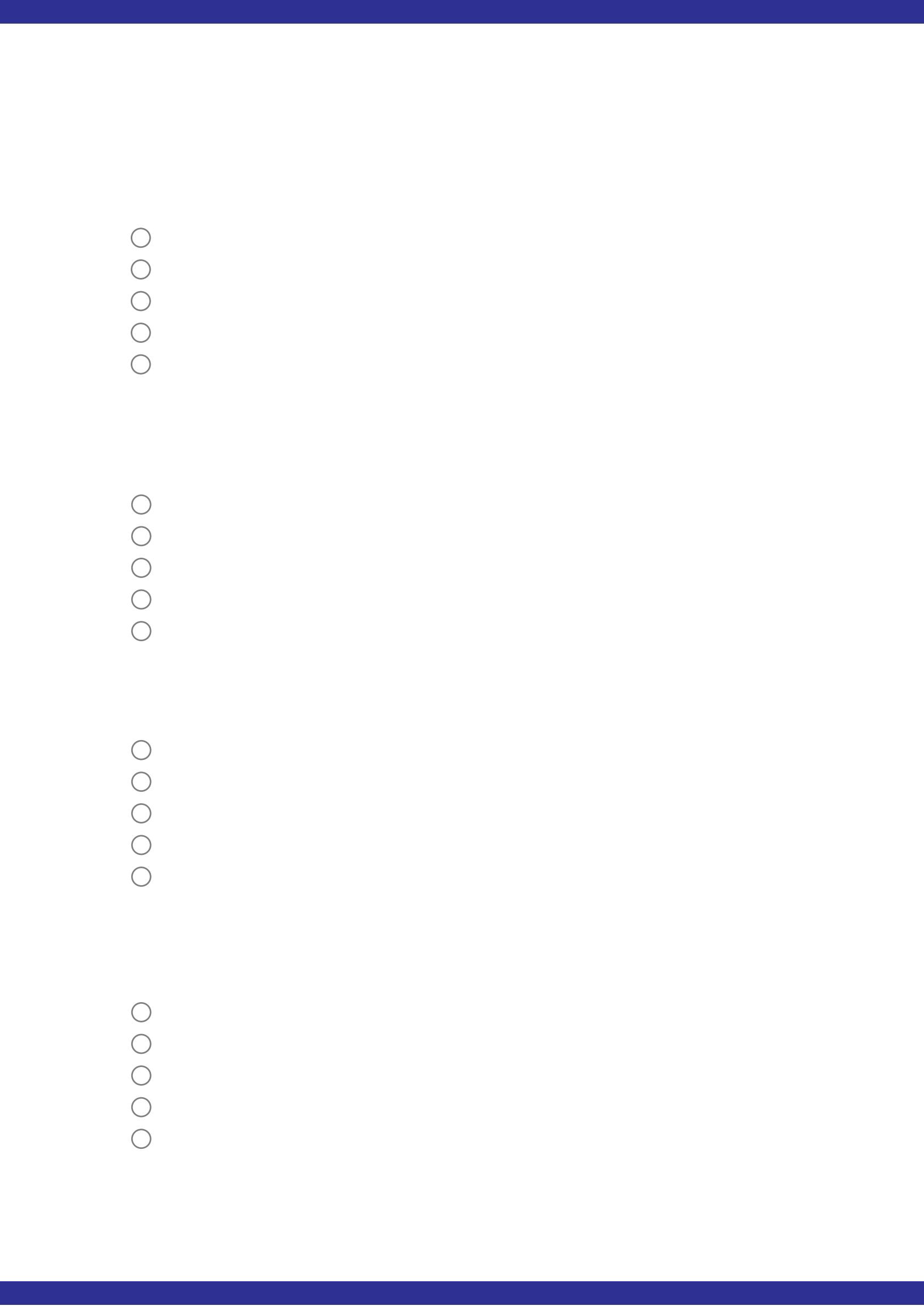
Machine Translated by Google

**SLEEP CHARACTERISTICS**

How often do you feel so sleepy that you have trouble paying attention in class?

Always, every day)

Frequently (3 or more times per week)

Almost never (less than once a week)

Sometimes (1 or 2 times a week) Never

How often do you fall asleep or get sleepy while doing homework?

Always, every day)

Frequently (3 or more times per week)

Almost never (less than once a week)

Sometimes (1 or 2 times a week) Never

Are you attentive or alert in class?

Always, every day)

Frequently (3 or more times per week)

Almost never (less than once a week)

Sometimes (1 or 2 times a week) Never

How often do you feel tired and in a bad mood during the day?

Always, every day)

Frequently (3 or more times per week)

Almost never (less than once a week)

Sometimes (1 or 2 times a week) Never


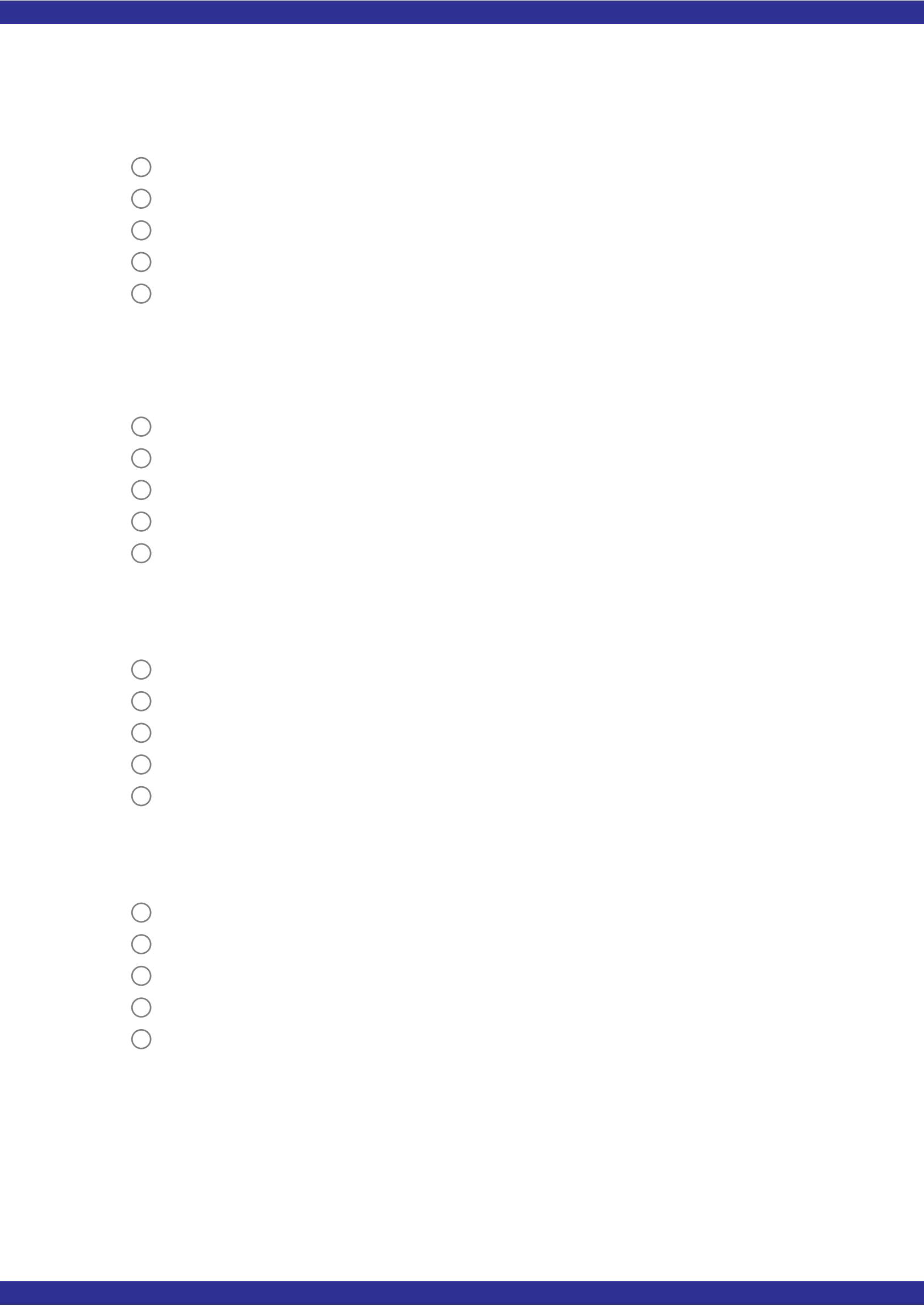
Machine Translated by Google

Do you have a hard time getting out of bed in the morning?

Always, every day)

Frequently (3 or more times per week)

Almost never (less than once a week)

Sometimes (1 or 2 times a week) Never

Do you fall asleep again after being woken up in the morning?

Always, every day)

Frequently (3 or more times per week)

Almost never (less than once a week)

Sometimes (1 or 2 times a week) Never

Do you need someone to wake you up in the morning?

Always, every day)

Frequently (3 or more times per week)

Almost never (less than once a week)

Sometimes (1 or 2 times a week) Never

How often do you feel the need to sleep longer?

Always, every day)

Frequently (3 or more times per week)

Almost never (less than once a week)

Sometimes (1 or 2 times a week) Never


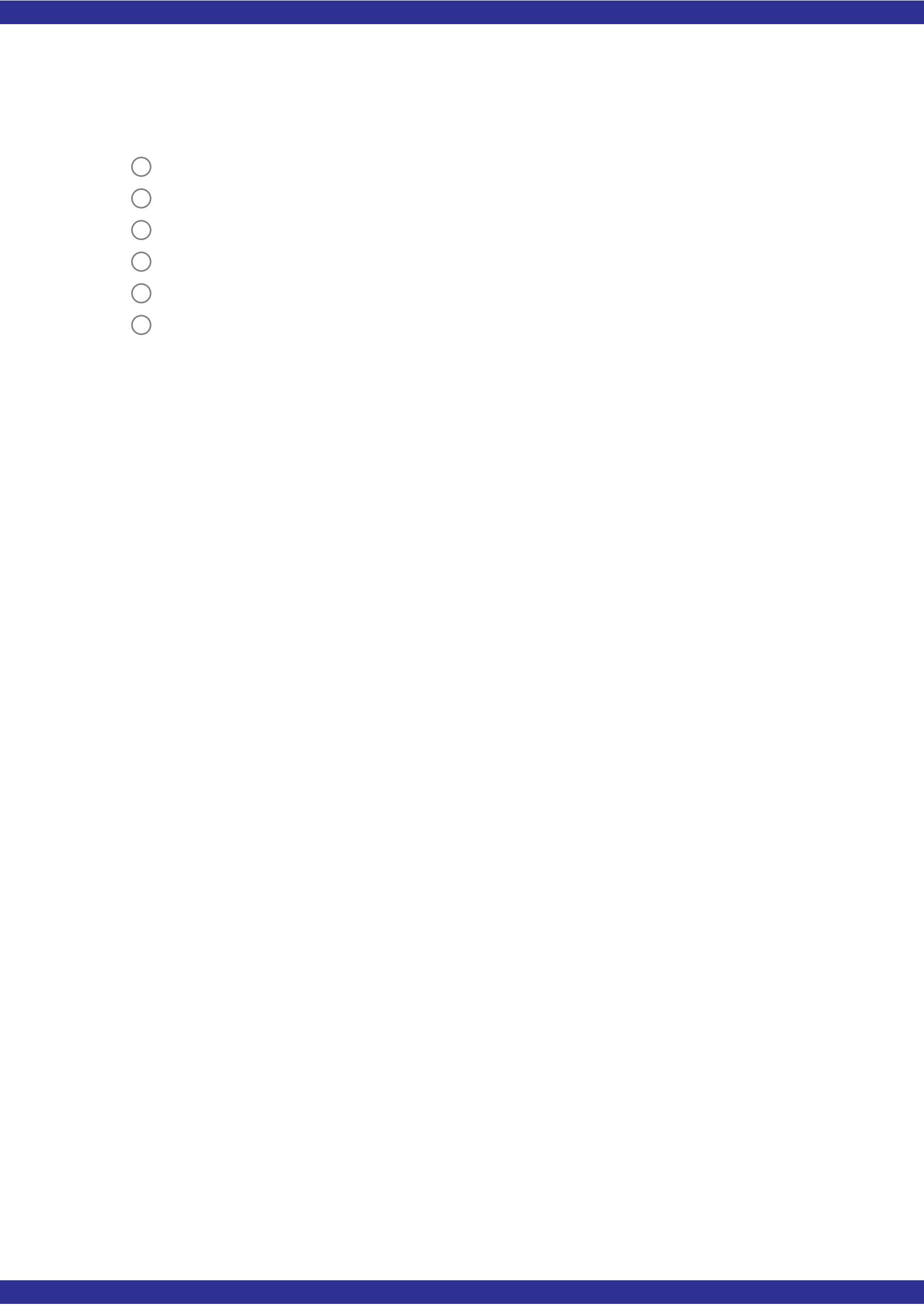
Machine Translated by Google

How do you travel to school most of the time?

Auto

Walking

School transportation

Public transport (bus, train, other)

Cycling

Others

How long does it take from the moment you leave your house until you arrive at school? (indicates in hours, minutes)

:

Hours minutes

How long do you nap on weekdays? (if you don't take a nap, dial 00:00)

:

Hours minutes

How long do you nap on weekends or holidays? (if you don't take a nap, dial 00:00)

:

Hours minutes

What time do you have dinner on weekdays?

:

Hours minutes

What time do you go to bed on weekdays?

:

Hours minutes

How long does it take you to fall asleep?

:

Hours minutes


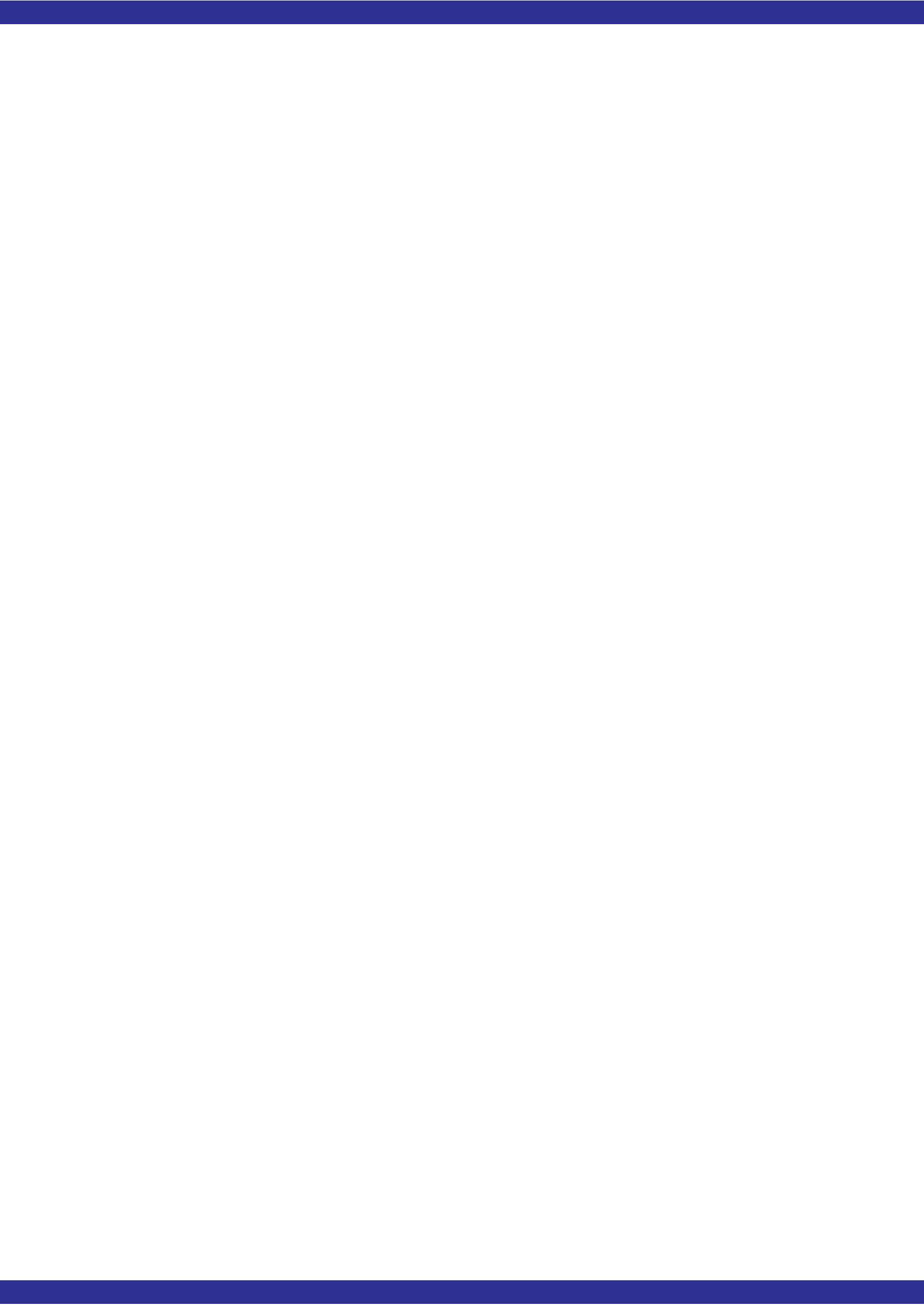
Machine Translated by Google

What time do you get up on weekdays?

:

Hours minutes

What time do you go to bed on weekends?

:

Hours minutes

What time do you get up on weekends?

:

Hours minutes

**Usually, outside of school hours and during the week.**

**How much time do you dedicate per day to the following games or activities? (if you do not, indicate time "00:00")**

Video games on computer or consoles (PlayStation, Xbox, WII, etc.).

:

Hours minutes

Games on tablet and/or cell phone.

:

Hours minutes

Use of social networks (facebook, instagram, twitter, snapchat, etc.)

:

Hours minutes

Watch television or online content (TV, Netflix, YouTube, etc.)

:

Hours minutes

Other activities in front of a screen.

:

Hours minutes


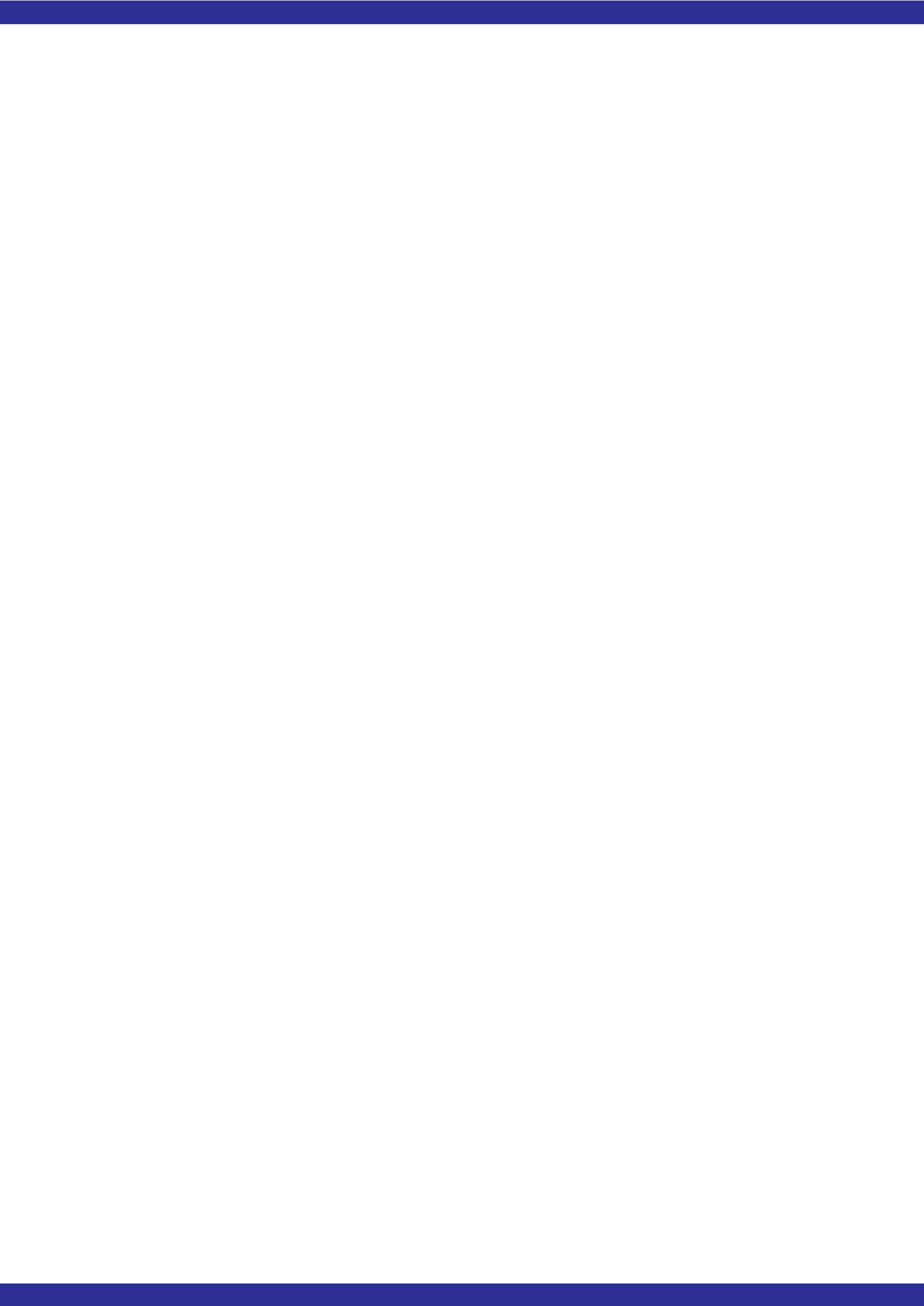
Machine Translated by Google

**In your bedroom and during the last hour before you fall asleep. How often do you use the following electronic devices?**

PC or laptop

:

Hours minutes

Cell phone

:

Hours minutes

tablets

:

Hours minutes

Game console

:

Hours minutes

TV or Smart TV

:

Hours minutes
